# Cerebral Venous Thrombosis at High Altitude: A Retrospective Cohort of Twenty-one Consecutive Patients

**DOI:** 10.7759/cureus.4940

**Published:** 2019-06-19

**Authors:** Nicolas K Khattar, Fitri Sumardi, Ajmal Zemmar, Quinghua Liang, Haiyang Li, Yazhou Xing, Hugo Andrade-Barazarte, Jack L Fleming, Iype Cherian, Juha Hernesniemi, Joseph S Neimat, Robert F James, Sunil Munakomi, Dale Ding

**Affiliations:** 1 Neurological Surgery, Juha Hernesniemi International Neurosurgery Center, Henan Provincial People's Hospital, Zhengzhou, CHN; 2 Neurological Surgery, Juha Hernesniemi International Neurosurgery Center, Henan Provincial People’s Hospital, Zhengzhou, CHN; 3 Neurological Surgery, Nobel Medical College and Teaching Hospital, Biratnagar, NPL; 4 Neurological Surgery, University of Louisville School of Medicine, Louisville, USA

**Keywords:** cerebral venous thrombosis, developing countries, patient management, low-molecular-weight heparin, gos

## Abstract

Background

Cerebral venous thrombosis (CVT) is a rare cerebrovascular disorder, comprising <1% of all strokes. The incidence of CVT is higher in females but a small number of cases suggest that men have a higher risk for CVT in high elevation. The aim of this retrospective cohort study is to investigate this gender-related relationship and to describe the baseline characteristics and treatment outcomes of patients who suffered CVT at high altitude in eastern Nepal.

Methods

We conducted a retrospective analysis of 21 consecutive patients with CVT at a tertiary care center in Nepal from July 2017 to January 2018. Clinical data, radiologic characteristics, therapeutic strategies, and outcomes were analyzed. The Glasgow Outcome Scale (GOS) at discharge was reported for each patient.

Result

The study cohort comprised 21 patients (76% males) with a mean of 56 years. Medical comorbidities included hypertension (76%) and diabetes mellitus (57%). All patients received low-molecular-weight heparin therapy (LMWH). Eight patients (38%) underwent decompressive craniectomy while the remaining 13 (62%) were treated with medical therapy alone. The GOS at discharge was 5 in 57%, 2-4 in 33%, and 1 in 10%.

Conclusion

In our series, men were found to have a higher risk for CVT at high altitude. The reversal in the gender ratio could be related to elevation, but could also be confounded by alcoholism. Increasingly sophisticated imaging techniques, such as computed tomography venography (CTV) and magnetic resonance venography (MRV), have facilitated the diagnosis of CVT. LMWH is a safe and easily accessible treatment option, especially in developing countries. Further studies are needed to assess the incidence and prevalence of CVT in the developing world, to establish the gender-related trends.

## Introduction

Cerebral venous thrombosis (CVT) is a rare cerebrovascular disorder, comprising 0.2%-1.3% of all strokes in the developed world [1-3]. There is limited literature describing the incidence or prevalence of CVT in developing countries, but it has been posited to be higher than in developed countries [4]. Nepal is one of the few countries in the world with a higher average altitude (10,000 ft). Increased incidence of CVT has been associated with high elevation [5-7], and strenuous activity at peak summits [6,8-11]. Most of the reports reported an increased incidence of CVT in men at high altitude. This is in stark contrast to the known increased risk of CVT in women at lower elevations [12]. The present series includes 21 patients and, to our knowledge, is the largest cohort of CVT patients at high altitude.

## Materials and methods

Patient selection

We retrospectively reviewed all patients presenting with clinical and radiographic evidence of cerebral venous sinus thrombosis treated at the Nobel Medical College and Teaching Hospital in Nepal between July 2017 and January 2018.

Neuroimaging evaluation and preoperative planning

Brain computed tomography (CT) was obtained in every patient prior to a definitive diagnosis. CT scanning was easier to perform and was more readily available than magnetic resonance imaging (MRI) upon admission. Upon finding evidence of intracerebral hemorrhagic infarction, a CT angiography/venography of the brain was subsequently obtained to confirm the putative diagnosis.

Baseline and follow-up data

Baseline data comprised patient demographics and clinical presentation. Patient demographics included age, gender, and medical comorbidities. Clinical presentation included symptoms and Glasgow Coma Scale (GCS) at presentation. Follow-up data included Glasgow outcome scale (GOS) at discharge. No further follow-up data was available.

Statistical analysis

Microsoft Excel 2016 (Microsoft Corporation) was used to collect and analyze data. Baseline and follow-up variables were analyzed using descriptive statistics. Categorical variables were presented as frequency and percentages, and continuous variables were presented as median and range.

## Results

The study cohort comprised 21 patients with CVT. The anatomical distribution of the thrombosis pattern was not available. Table 1 summarizes the baseline characteristics and outcomes of the study cohort.

**Table 1:** Baseline characteristics, treatment strategies and outcomes

	Number of patients (%)	
Age Group (years)		
20-40		8 (38)
50-70		12 (57)
>70		1 (5)
Sex		
Male		16 (76)
Female		5 (24)
Co-morbidities		
Alcohol abuse		16 (76)
Oral contraceptives		3 (14)
Trauma		2 (10)
Clinical presentation		
Headache		9 (43)
Dizziness		5 (24)
Seizure		4 (19)
Dysarthria		3 (10)
Duration from onset of symptoms (hours)		
<12		18 (86)
>12		3 (14)
Glasgow Coma Score		
<8		2 (10)
9-12		7 (33)
>12		12 (57)
Treatment plan		
Conservative		13 (62)
Operative		8 (38)
Glasgow Outcome Scale (GOS) at discharge		
1		12 (57)
2-4		7 (33)
5		2 (10)

Baseline characteristics

The median age of patients was 56 years (range 61-87 years) and 76% were male. The clinical presentations were headaches (43%), dizziness (24%), seizure (19%), and dysarthria (10%). Medical comorbidities included alcoholism in 16 patients (76%), oral contraceptive use in three (14%), and a history of trauma in two (10%). The GCS at presentation was >12 in 12 patients (57%), 9-12 in seven (33%), and <8 in two (10%). Eight patients (38%) had a midline shift and cerebral edema at presentation. All patients presented with evidence of hemorrhagic conversion on the initial brain CT.

Hospital course and follow-up outcomes

Thirteen patients (62%) had normal prothrombin time (PT), partial thromboplastin time (PTT) and D-dimer levels. Eight patients (38%) showed mild thrombocytosis with decreased PT/PTT in a non-specific pattern. Thirteen patients (62%) were treated conservatively with therapeutic low-molecular-weight heparin (LMWH) for a minimum of five days. None of the patients treated conservatively with LMWH showed any progressions of the hemorrhages on brain CT. The eight patients (38%), who initially presented with evidence of cerebral edema, increased intracranial pressure and midline shift were treated with a decompressive craniectomy with or without hematoma evacuation. The median length of hospital stay was 14 days. Twelve patients (57%) were discharged home from the hospital with mild or no neurological disability (GOS 5) [9]. Seven patients (33%) had discharge outcomes ranging from a vegetative state to severe disability (GOS 2-4). Two patients died from CVT (GOS 1; mortality rate 10%).

Case example

**Figure 1:**
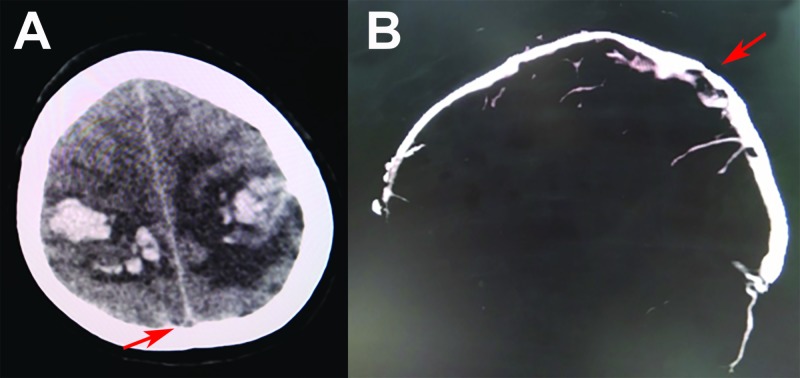
Initial radiographic evidence of cerebral venous thrombosis (CVT) A) Initial brain computed tomography (CT) showing bilateral hemorrhagic infarctions with a non-specific distribution; the empty delta sign is observed (arrow); B) CT venography shows evidence of thrombus in the posterior superior sagittal sinus (arrow).

A 38-year-old man with a baseline modified Rankin scale (mRS) of 0 and severe alcoholism presented with a severe headache. Brain CT showed bilateral intracerebral hemorrhages (Figure 1A). The patient then underwent CT venography, which showed partial thrombosis of the superior sagittal sinus (Figure 1B). He was treated with a decompressive craniectomy for relief of intracranial hypertension. The patient was subsequently started on LMWH. Unfortunately, the patient’s condition did not improve postoperatively and he expired a few days later.

## Discussion

CVT continues to be an often overlooked or delayed diagnosis due to the wide range of presenting clinical symptoms, the most common of which is headache [1,10-11]. Headaches may develop acutely and are clinically indistinguishable from headaches in patients with subarachnoid hemorrhage. The time of onset of headache i is usually less than 12 hours from the diagnosis of CVT [2-3]. In our series, headache was the presenting symptom in 43%. CVT should always be considered in the differential diagnosis of patients who present with focal neurological deficits, seizures or altered mental status. CVT has been attributed to various causes related to structural obstruction of the cerebral venous sinuses or increased blood viscosity.

The incidence of CVT is documented to be three times higher in women compared to men in the young to middle-aged adult age group [4]. Gender-specific skewing is generally attributed to female-specific hyperviscosity state risk factors including oral contraceptives, pregnancy, puerperium or hormone-replacement therapies leading to a hypercoagulable state [9]. Other causes include systemic hypercoagulable state, e.g., in neoplastic disease and paraneoplastic syndromes as well as inflammatory and autoimmune diseases [10-11]. Furthermore, structural obstruction of the cerebral venous sinuses include traumatic compression, direct injury due to fractures or compression from mass lesions including tumors, arteriovenous malformations, and dural arteriovenous fistulae [12-14]. This gender-ratio is reversed at high altitude [5-7]. While this observation was reported in single case reports or small case series, our study confirms this phenomenon in the yet largest published series of 21 patients. Physical activity and physiological alterations at high altitude could differentially be causing the increased risk in men [15-18]. In developing countries, the incidence of CVT has been observed to be higher than the range reported in the literature from the developed world [4]. Our cohort represents a series of CVT patients from Nepal, an Asian country with a significantly higher average altitude. Even though estimating the incidence of CVT in all patients presenting with an intracranial hemorrhage is challenging, it is worth noting that all cases in our study cohort were accrued in a six-month period. In our series, male patients presenting with CVT at high altitude also had higher rates of alcoholism, which could be a confounding factor.

Patents in our series were diagnosed with CVT via brain CT or CT angiography [19]. Non-contrast brain CT remains the first diagnostic study in cases of lobar intraparenchymal hemorrhages or infarctions that do not follow an arterial distribution [20-21]. However, the sensitivity of a non-contrast brain CT is variable (40%-70%). Non-invasive imaging using intravenous contrast agents significantly increases the sensitivity of diagnosis, with magnetic resonance venography (MRV) as the most sensitive diagnostic imaging modality [21-22]. CT venography is a reasonable alternative to MRV for the diagnosis of CVT [20]. In a recent review, CT and MR were found to be of equivalent sensitivity and specificity in the diagnosis of CVT, and the utilization of either imaging modality would then depend on availability [7].

The management of patients with CVT depends upon their clinical condition upon arrival to the hospital [1]. Initial presentation with extensive hemorrhages associated with signs of elevated intracranial pressure, midline shift or impending brain herniation warrants urgent surgical intervention with decompressive craniectomy [1,20,23]. Anticoagulation with intravenous heparinization is the mainstay of medical treatment when the patient is neurologically stable [20,24]. A randomized controlled trial (RCT) of 66 patients found that patients treated with LMWH (N=34) had a significantly lower rate of in-hospital mortality than unfractionated heparin (N=32). The favorable effect of LMWH in the RCT is likely related to suboptimal anticoagulation and the need for coagulation monitoring [25]. Therefore, LMWH is a reasonable alternative to unfractionated heparin for the treatment of CVT, unless the patient has a contraindication for the former therapy, such as compromised renal function or need for reversal for a neurosurgical intervention. In our cohort, all patients received therapeutic anticoagulation in the form heparin infusions once the diagnosis of CVT was confirmed. The heparin infusion was reversed prior to any surgical interventions. Prophylactic subcutaneous heparin was administered on post-operative day one and the patient was subsequently transitioned to a therapeutic heparin infusion 48 hours later. There was no evidence of increased intracranial hemorrhage or emergence of new hemorrhagic lesions after initiation of anticoagulation. Recent reports have shown that endovascular mechanical thrombectomy could have a role in the treatment of CVT refractory to medical management [26-27]. However, such therapy may not always be available in areas like Eastern Nepal.

Limitations

The relatively small size of the study cohort and the short follow-up time point of six months preclude any meaningful statistical analyses, particularly with regard to identifying predictive factors of CVT or specific patterns within the population of Nepal. Additionally, the retrospective design of the study subjects our findings to selection and treatment biases. Medical record keeping remains a challenge in developing countries, which limits the granularity of clinical data and follow-up. Additional studies comprising larger patient cohorts with more detailed baseline and outcomes data are needed to validate our findings.

## Conclusions

CVT remains a challenging diagnosis in developing countries. In our series, the incidence of CVT was significantly higher in men at high altitude. Even though this could indicate an intrinsic higher risk for men at higher elevation, there could be other confounding factors and future studies should seek to better characterize the risk factors of CVT in developing countries, such as Nepal. With the increasing sensitivity of imaging techniques, such as CT venography or MRV, the diagnosis of CVT has been facilitated in centers where these modalities are available. Our preliminary findings show that LMWH is a safe and effective therapy for patients with CVT in developing countries. Additional data regarding treatment patterns and long-term outcomes are also necessary to establish a management algorithm that can be tailored to the available therapies in each of these unique populations.
